# Supplementary material for: Serum Biomarker Concentrations upon Admission in Acute Traumatic Brain Injury: Associations with TBI Severity, Toxoplasma gondii Infection, and Outcome in a Referral Hospital Setting in Cameroon
Source: NeuroSci. 2023 Jul 3;4(3):164–77. doi: 10.3390/neurosci4030015 (PMC11523680; doi:10.3390/neurosci4030015)
Supplement: Supplementary file 1 [file neurosci-04-00015-s001.zip › neurosci-2441591-supplementary.pdf]

Supplementary Table S1: Prevalence of Toxoplasma gondii infection in TBI according to sociodemographic characteristics.

Characteristic	Overall N = 160	Negative N = 108	Positive N = 52	P-value
**Age**	32 (26, 39)	32 (27, 38)	30 (23, 39)	0.6
**Gender**				0.072
Female	16 (10%)	14 (13%)	2 (3.8%)	
Male	144 (90%)	94 (87%)	50 (96%)	
**Profession**				0.5
Bike riders	43 (27%)	28 (26%)	15 (29%)	
Employee in service	30 (19%)	21 (19%)	9 (17%)	
Manual workers	24 (15%)	16 (15%)	8 (15%)	
Others	26 (16%)	15 (14%)	11 (21%)	
Student	16 (10%)	10 (9.3%)	6 (12%)	
Unemployed	21 (13%)	18 (17%)	3 (5.8%)	
**Classification of TBI**				
Mild		42 (39%)	24 (46%)	0.7
Moderate		38 (35%)	17 (33%)	
Severe		28 (26%)	11 (21%)	

Median (IQR); n (%) *Wilcoxon rank sum test; Pearson's Chi-squared test*

TBI: Traumatic brain injury
